# Weak D prevalence among Indian blood donors

**DOI:** 10.4103/0973-6247.67030

**Published:** 2010-07

**Authors:** R. N. Makroo, Vimarsh Raina, Mohit Chowdhry, Aakanksha Bhatia, Richa Gupta, N.L. Rosamma

**Affiliations:** Department of Transfusion Medicine, Indraprastha Apollo Hospitals, Sarita Vihar, New Delhi-110076, India

Sir,

The field of medicine has since infinity been associated with consistent discoveries thus leading to ever increasing list of advancing technologies. When talking about transfusion medicine, the discovery of blood group antigens marked the revolutionising step in the success of blood transfusions. Following the discovery of ABO antigens by Landsteiner in 1901,[1] the next most important discovery was that of Rh antigens in 1939 by Landsteiner and Weiner further leading to description of haemolytic disease of new born by Levine and Stetson.[2]

The Rh antigen itself has been studied extensively and its alleles recognised. The Rh gene lies on chromosome number 1 and is carried in groups of three.[3] The Rh locus is composed of two highly homologous genes: the *RHD* gene, which encodes the D protein, and the *RHCE* gene, which encodes the C, c, E, and e proteins.[4] 6 alleles have been identified (c, C, e, E, d, D) as against only 5 antigens (c, C, e, E, D), d being an amorph gene.[5] Among these, D is the most immunogenic. Consequently, D often is called the Rh antigen, and the terms Rh+ and Rh– refer, respectively, to the presence or absence of D antigen.

The Rh complex is critical to the structure of the RBC membrane. Rh null erythrocytes, which lack Rh proteins, are stomatocytic and spherocytic, and affected individuals have haemolytic anemia.[6]

The RhD antigen has been reported to consist of a mosaic of at least 9 D epitopes (epD1-epD9).[7] Recent work involving testing a large number of monoclonal anti-D (MAb-D) reagents has suggested the presence of a minimum of 30 different epitope structures[8] distributed along the extracellular portions of the RhD protein. Thus a change, or changes, in the amino acid sequence of RhD may not ablate the entire D antigen but can cause epitope loss, giving rise to variant forms of D antigen.

“*Weak D*” RBCs demonstrate reduced quantities of the D antigen. As a result weak or no agglutination reaction is demonstrated by these RBCs with the anti D reagents at the immediate spin phase. About 0.1 to 2 percent of white Caucasians have this Rh phenotype.[9] Missense mutations observed in the alleles of all weak D types have been demonstrated to be the probable cause for the reduced antigen D expression in these cases.[10]

In “*Partial D*” RBCs, the RHD protein is mutated in an exofacial loop, eliminating at least one D-specific epitope. However, the numbers of RhD antigens on the RBC surface are normal.[10] The “*DEL*” phenotype is a third group of D variants. DEL cannot be detected by routine serological testing. It is, however, easily detected by genetic analysis.[11] DEL RBCs contain an extraordinarily low number of D antigens but, despite this paucity, can cause primary[12] and secondary[13] immune responses against the D antigen in D negative recipients. Fortunately its incidence is very low. It is found mainly amongst Asian populations where a recent study found a DEL allele in approximately 13% to 16% of serologically D negative Chinese and Japanese individuals.[14] A myriad of different serological techniques and reagents, each with different sensitivities, and a rapidly expanding understanding of the genetics of the *RHD* gene have greatly complicated D typing.

In this study we tried to estimate the prevalence of weak D in our population based on the serological techniques. As ours is a prominent tertiary care hospital of the country, the donors here come from various parts of the country and are representative of the whole Indian population.

This study was conducted in the Department of Transfusion Medicine, Indraprastha Apollo Hospitals from 01 January 1999 to 31^st^ December 2008. During this period, a total of 1, 84,072 donors came to our blood bank. As a routine protocol, Rh typing was done for all these donors. Those who tested negative for Rh D antigen were further subjected to weak D testing.

Routine Rh typing was done using the immediate spin tube technique with two anti-D reagents (*RHOFINAL Anti D IgM + IgG by TULIP and RHESOLVE Anti D IgM monoclonal by ORTHO*). Blood samples, which were negative for agglutination by immediate spin tube method, were further tested for weak D.

A 5% suspension of the cells to be tested was made. Three tubes were taken and labelled as test, negative and positive control. To the tube labelled as test 2 drops of Anti D serum (RHOFINAL Anti D IgM + IgG by TULIP) was added. 2 drops of 22% bovine albumin were added to the negative tube and to the positive control 2 drops of weak anti D (i.e. 1 drop of *Anti D IgM monoclonal by ORTHO +* 19 drops of saline). 2 drops of 5% cell suspension was added to all the test tubes. They were mixed and incubated at 37°C in a water bath for 45-60 minutes (as per manufacturer’s instructions).

The tube was gently re-suspended and the cell button observed for agglutination. If the test red cells were agglutinated (but not in the negative control tube) the test was recorded as positive. If the test cells were not agglutinated or the results were doubtful, the cells were washed three to four times with normal saline. After the final wash, the saline was decanted and one to two drops of antiglobulin serum was added according to manufacturer’s instructions. Following this, the contents of the test tube were mixed and the tube was centrifuged at 1000 rpm for 30 seconds. The cell button was then gently re-suspended and examined for agglutination. All negative results were confirmed under the microscope. Samples showing agglutination after incubation or after addition of AHG serum (Bioclone Anti IgG, C3d by Ortho) were considered to be weak D positive. The data was analysed to provide the following results.

**Figure 1 a-b:**
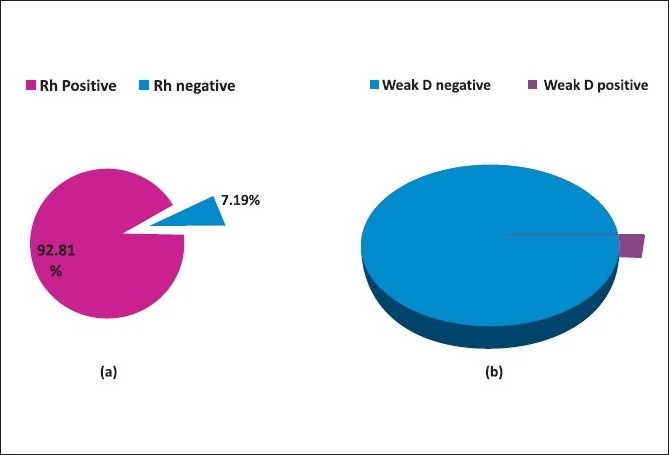
Data label (value) missing in diagram b, 0.12% (pink region)

A total of 1, 84, 072 donors were studied. Among these 13, 253 (7.2%) were tested to be Rh negative. All the Rh negative samples were subjected to weak D testing. Of the 13, 253 samples that tested to be Rh negative, 16 (0.01% of total donors and 0.12% of Rh negative donors) were found to be weak D positive. [Table 1, Figure 1a, and b]

**Table 1:** Weak D prevalence in blood donors

	Number	Percentage
Total donors	1,84,072	
Rh positive	1,70,819	92.81
Rh negative	13,253	7.19
Weak D positive	16	0.01

In our study 7.19% of the donors were Rh negative and the weak D variant was detected in about 0.01%. This extensive study was done by us over a period of 10 years. To our knowledge, this study is the largest of its kind that we came across in the published literature. Our hospital, being a major tertiary care centre of the country, caters to a population covering large geographical areas and therefore our results can be considered as representative of the whole country.

Rh system is the most complex of all 29 blood group systems. New discoveries relating to the *RHD* gene, and an appreciation of its variant phenotypes have challenged the way that D status is assigned to both blood donors and blood product recipients. The weak D, previously called D^u^, has been the subject of many studies ever since it was identified. Controversy as to whether the weak D is a quantitative or qualitative D variant and whether or not it stimulates anti-D immunization in RhD-negative persons still persists.

Historically, RBCs that react with anti-D only after extended testing in the AHG phase are called weak D. However, the number of samples classified as weak D depends on the characteristics of the typing reagent.[15] The improved sensitivities of the anti D sera have decreased the prevalence of weak D phenotypes. The prevalence also varies from region to region. We reported Weak D in 0.01% of our donor population. Slightly lower values have been reported in a Korean study and higher values have been reported in the Caucasians.[16,17]

The main concern about this Rh phenotype arises due to the risk of alloimmunisation among the recipients. Since, the “D” antigen is highly immunogenic, individuals with the Weak D phenotype are designated D pos. The patients with weak D are considered D negative and must be transfused with D negative blood. Mothers with weak D fetus must receive Rh immunoprophylaxis[18] as passage of Weak D red cells from fetus to mother may cause sensitization.

However, there have been studies suggesting that the most common weak D types (1, 2, 3, 4.0, and 4.1), encompassing more than 90% of all European weak D individuals, do not appear to be susceptible to immunization to the D antigen[7] These individuals could safely receive D positive blood and do not need Rh immune globulin prophylaxis during pregnancy. However, serological tests cannot discriminate between these weak D types and those that are susceptible to alloimmunization; only a molecular analysis of the *RHD* gene can distinguish between weak D types.

The prevalence of Rh negativity in our donor population has been estimated to be 7.19% and that of weak D antigen 0.01% in our study. However, a study analysing repeat antibody screens of serologically D negative patients with weak D alleles who have been exposed to D+ RBCs is needed to quantify the absolute risk of sensitization.
